# P-463. Diversity of KSHV Subtypes in People Living with HIV in a Large Urban Center in Dallas, Texas

**DOI:** 10.1093/ofid/ofae631.662

**Published:** 2025-01-29

**Authors:** Sheena Knights, Vickie A Marshall, Nazzarena Labo, Wendell Miley, Elena Cornejo Castro, Susana Lazarte, Elizabeth Chiao, Denise Whitby, Ank E Nijhawan

**Affiliations:** University of Texas Southwestern Medical Center, Dallas, Texas; Frederick National Laboratory for Cancer Research, Frederick, Maryland; Frederick National Laboratory for Cancer Research, Frederick, Maryland; Frederick National Laboratory for Cancer Research, Frederick, Maryland; Frederick National Laboratory for Cancer Research, Frederick, Maryland; UT Southwestern Medical Center, Dallas, TX; University of Texas MD Anderson Cancer Center, Houston, Texas; Frederick National Laboratory for Cancer Research, Frederick, Maryland; UT Southwestern, Dallas, Texas

## Abstract

**Background:**

Human herpesvirus-8 (HHV-8), also known as Kaposi’s sarcoma-associated herpesvirus (KSHV), causes Kaposi’s sarcoma (KS) and other KSHV-associated disease (KAD). We previously described high KSHV seroprevalence and high KS incidence in a population of men who have sex with men (MSM) with HIV in Dallas, Texas. We now describe viral genetics in the same population.

**Table 1:**
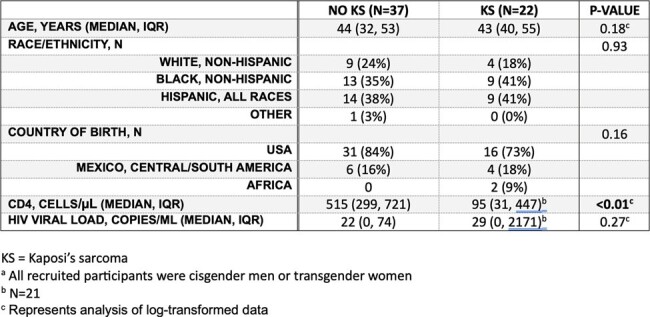
Participant Characteristics

**Methods:**

We analyzed samples from participants recruited in 2 different studies, one enrolling MSM with HIV with and without KAD, and the other enrolling MSM with HIV and KAD. We measured KSHV IgG in serum using a combination of ELISA and a bead-based multiplex assay. Oral fluid samples of all individuals with KAD and seropositive individuals with no KAD were analyzed by RT-PCR to detect and quantify KSHV DNA. Samples with detectable KSHV underwent Sanger and/or next generation sequencing to determine K1 subtype.

**Figure 1:**
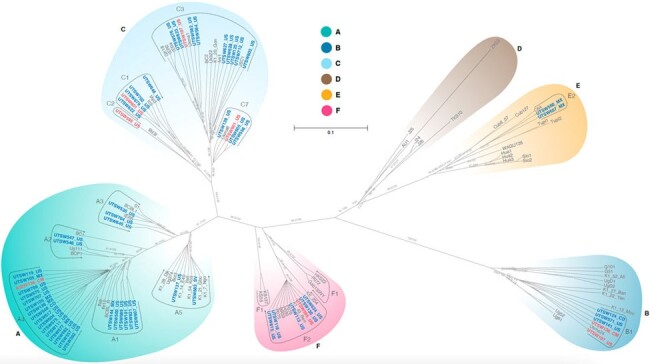
Phylogenetic Tree of Participant Samples

**Results:**

Overall, 281 participants were recruited; 59 shed enough virus for K1 subtyping. Of these, 37 did not have KAD, and 22 had KAD, all had KS. **Table 1** describes the demographic characteristics of these participants. Notably, those with KS had lower median CD4 countsthan those without KS (p < 0.01). All known K1 subtypes, except for D, were identified in this cohort, including the rare E and F subtypes. The latter, which only recently had been described outside Africa was identified in 6 individuals. Four participants had mixed infections with 2 or more KSHV subtype (**Figure 1**). **Figure 2** illustrates clinical presentations by KSHV subtype. Limitations in sequencing technology and study design permit no inferences on possible associations between KSHV subtype and KS onset or severity. We observed a larger proportion of subtypes B and F, typical of African populations, in participants with KS, than those with no KAD (8/27 subtypes detected vs 3/37). However, A and C were the most commonly identified subtypes in KS (19/27 subtypes in those with KS vs 32/37 subtypes in those without KS), including in advanced cases (i.e. T1 stage, 10/13).

**Figure 2:**
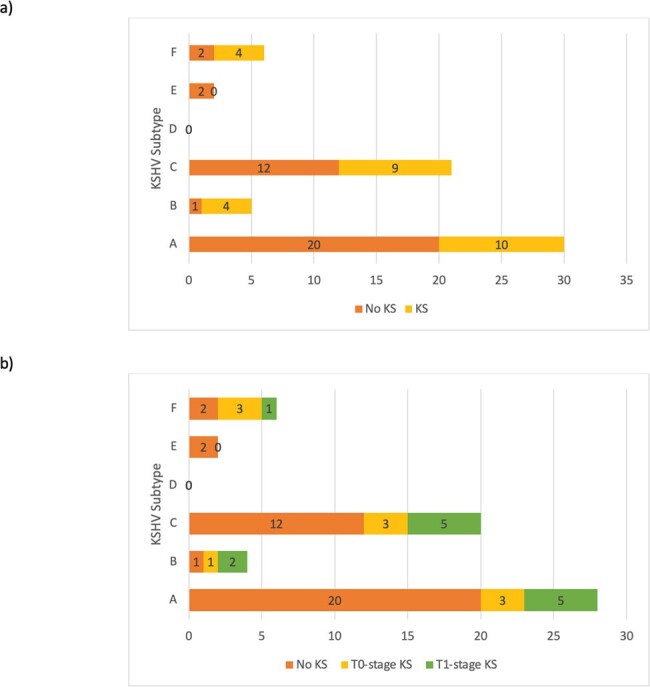
KSHV Subtypes and Clinical Presentation

**Conclusion:**

In conclusion, we observed extreme diversity in KSHV genomes sequenced in a single institution in Dallas, TX. Further studies are needed to better understand the relationship between viral genetics and KSHV epidemiology and disease risk in the southern US.

**Disclosures:**

**Ank E. Nijhawan, MD, MPH, MSCS**, Gilead Sciences: Grant/Research Support

